# *Mycolicibacterium fortuitum* genomic epidemiology, resistome and virulome

**DOI:** 10.1590/0074-02760210247

**Published:** 2022-01-10

**Authors:** Sergio Morgado, Nilcéia de Veiga Ramos, Fernanda Freitas, Érica Lourenço da Fonseca, Ana Carolina Vicente

**Affiliations:** 1 Fundação Oswaldo Cruz-Fiocruz, Instituto Oswaldo Cruz, Laboratório de Genética Molecular de Microrganismos, Rio de Janeiro, RJ, Brasil; 2 Universidade de Vassouras, Faculdade de Ciências Médicas de Maricá, Maricá, RJ, Brasil

**Keywords:** arr, rifampin, resistance, ESX, type VII secretion system, opportunist pathogen

## Abstract

**BACKGROUND:**

*Mycolicibacterium fortuitum* is an opportunistic pathogen associated with human and animal infection worldwide. Studies concerning this species are mainly represented by case reports, some of them addressing drug susceptibility with a focus on a specific geographic region, so there is a gap in relation to the global epidemiological scenario.

**OBJECTIVES:**

We aimed determine the global epidemiological scenario of *M. fortuitum* and analyse its traits associated with pathogenicity.

**METHODS:**

Based on publicly available genomes of *M. fortuitum* and a genome from Brazil (this study), we performed a genomic epidemiology analysis and *in silico* and *in vitro* characterisation of the resistome and virulome of this species.

**FINDINGS:**

Three main clusters were defined, one including isolates from the environment, human and animal infections recovered over nearly a century. An apparent intrinsic resistome comprises mechanisms associated with macrolides, beta-lactams, aminoglycosides and antitubercular drugs such as rifampin. Besides, the virulome presented Type VII secretion systems (T7SS), including ESX-1, ESX-3, ESX-4 and ESX-4-bis, some of which play a role on the virulence of *Mycobacteriaceae* species.

**MAIN CONCLUSIONS:**

Here, *M. fortuitum* was revealed as a reservoir of an expressive intrinsic resistome, as well as a virulome that may contribute to its success as a global opportunist pathogen.

*Mycobacteriaceae* comprehends a wide spectrum of environmental and pathogenic bacteria that eventually arise in clinics affecting human and animal health. Recently, members of this family have been reclassified into new genera, and thus rapid-growing species, such as *Mycobacterium fortuitum*, now belong to the *Mycolicibacterium* genus.^1^ *Mycolicibacterium fortuitum* is ubiquitous in the environment and its role as a pathogen is being recognised worldwide.^2^ In fact, the burden of disease due to non-tuberculous mycobacteria (NTM) may be underestimated, as infections by these organisms are not on the reportable list.^3^ In addition, NTM infections are increasing globally in humans and animals, and *M. fortuitum* is among the most prevalent NTM species enrolled in this scenario.^4^ ^,^ ^5^ ^,^ ^6^ *M. fortuitum* infections have been reported with a high prevalence of resistance to several drugs, including macrolides, beta-lactams, aminoglycosides and tetracyclines, in addition to antitubercular drugs (e.g., isoniazid, rifampin, ethambutol, clofazimine, ethionamide, and rifabutin).^7^ ^,^ ^8^ ^,^ ^9^ So far, studies of *M. fortuitum* are mainly represented by case reports in humans and animals, some of them addressing drug susceptibility with a focus on a specific geographic region, so there is a gap in relation to the global epidemiological scenario and systematic analysis of *M. fortuitum* traits associated with pathogenicity*.* The availability of *M. fortuitum* genomic information and metadata associated with these organisms (e.g., host, geographic location, year) is an opportunity to begin to fill the gap in the general biological characteristics of this zoonotic and widespread opportunistic bacteria.

## MATERIALS AND METHODS

*Genome sequences analysed* - A total of 25 *M. fortuitum* genomes were retrieved from the NCBI site (https://www.ncbi.nlm.nih.gov/genome/browse/#!/prokaryotes/14575/) in June 2021 (Table).

*Genome sequencing and assembly -* Genomic DNA of *M. fortuitum* 7G strain was extracted using NucleoSpin Microbial DNA (Macherey-Nagel) and sequenced with Nextera XT library kit on the Illumina HiSeq 2500 platform, generating 2 x 250 bp paired-end reads. The raw reads were submitted to quality control by NGS QC Toolkit v2.3.3^10^ and the genome was assembled using SPAdes v3.14.1.^11^ The genome and the raw reads were deposited at NCBI under the accession numbers JAEQRQ000000000 and SRR15257947, respectively.

*Drug susceptibility testing and cloning* - *M. fortuitum* 7G strain was grown in trypticase soy agar (TSA) medium supplemented with 0.05% Tween-80 (Sigma-Aldrich) at 22ºC for 72 h. Drug susceptibility of the *M. fortuitum* 7G strain was evaluated by E-test method (bioMerieux) in Mueller-Hinton agar plates for various drugs: azithromycin, clarithromycin, streptomycin, tobramycin, meropenem, cefalotin, cefepime, and rifampicin. The *arr* and *rox* polymerase chain reaction (PCR) products were cloned into the pGEM T-Easy Cloning Vector System (Promega) and used to transform *Escherichia coli* DH5α lineage (rifampicin MIC of 4 µg/mL).

*Phylogenetic analysis* - The *M. fortuitum* genomes were annotated using Prokka v1.14.6^12^ and submitted to Roary v3.13.0^13^ to determine the core genome. The single-nucleotide polymorphism (SNP) sites of the concatenated core genes (180,276 bp) were obtained using snp-sites v2.5.1.^14^ A phylogenetic neighbor joining tree was generated using PhyML v3.1 in Seaview v4^15^ with 1000 bootstrap replicates.

*Resistome and virulome analysis* - *M. fortuitum* genomes were surveyed for antibiotic resistance and virulence genes through ABRicate (https://github.com/tseemann/abricate) based on The Comprehensive Antibiotic Resistance Database^16^ and Virulence Factor Database.^17^ In addition, T7SS was searched based on the identification of the T7SS core proteins^18^ using HMMer package v3.1b2.^19^

## RESULTS AND DISCUSSION

Here, based on 25 *M. fortuitum* genomes (drafts and completes) available in the NCBI database and a sequenced genome from Brazil (this study; accession number JAEQRQ000000000), we performed a genomic epidemiology analysis and *in silico* and *in vitro* characterisation of the resistome^20^ of this species. This is a diverse set of *M. fortuitum* genomes considering the occurrence of the strains in terms of space, time, and origin, since they were isolated from environments, animals, and humans in several countries for almost a century (1923-2020). A core genome SNP analysis generated a neighbor-joining tree that revealed three main clusters (Figure). Based on the available metadata, it was possible to associate clusters I and III with isolates from different countries, while cluster II is composed only of isolates from South Africa. All isolates from cluster I (India, Mozambique, and Cambodia; 2008-2012) and II (South Africa; 2011-2012) were from human infections. Cluster III is quite diverse with isolates from human and animal infections, as well as from the environment, being recovered over almost a century (1923 to 2020) (Table).

**Figure:**
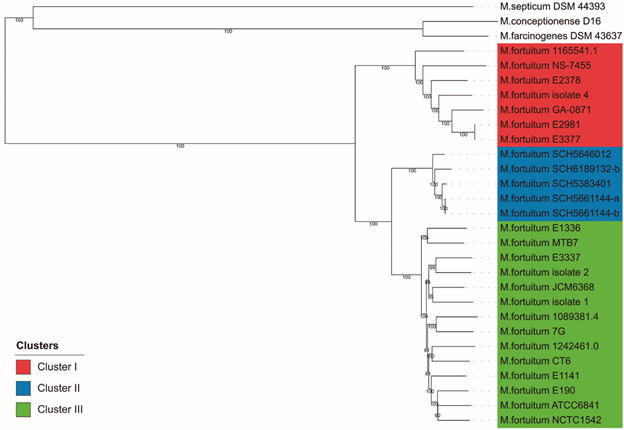
Single-nucleotide polymorphism (SNP)-based tree of 26 *Mycolicibacterium fortuitum* genomes highlighting the three main clusters.

**TABLE:** Metadata and resistome of the *Mycolicibacterium fortuitum* genomes

Genome/strain	*aph*(3’’)-Ic	*arr*-1	*bla*F	*erm*39	*aac*(2’)-Ib	*rbp*A	*tap*	*rox*	*sul*1	T7SS	Country	Year	Source	Cluster	Accession number
E2981	X	X	X	X	X	X	X	X		X	Cambodia	2011	ND	I	LZKN00000000
E3377	X	X	X	X	X	X	X	X		X	Cambodia	2011	ND	I	LZSI00000000
GA-0871	X	X	X	X	X	X	X	X		X	India	2008	human	I	MBER00000000
isolate_4	X	X	X	X	X	X	X	X		X	China	2013	human	I	JAAZWL000000000
E2378	X	X	X	X	X	X	X	X		X	Cambodia	2010	ND	I	LZKM00000000
1165541.1	X	X	X	X	X	X	X	X		X	Mozambique	2012	human	I	LZLP00000000
NS-7455	X	X	X	X	X	X	X	X		X	India	2010	human	I	MBEK00000000
SCH5383401	X	X	X	X	X	X	X	X		X	S. Africa	2012	human	II	LZIH00000000
SCH5661144-a	X	X	X	X	X	X	X	X		X	S. Africa	2012	human	II	LZSR00000000
SCH5661144-b	X	X	X	X	X	X	X	X		X	S. Africa	2011	human	II	LZSS00000000
SCH6189132-b	X	X	X	X	X	X	X	X		X	S. Africa	2012	human	II	LZIP00000000
SCH5646012	X	X	X	X	X	X	X	X		X	S. Africa	2012	human	II	LZSN00000000
MTB7	X	X	X	X	X	X	X	X		X	Morocco	2016	human	III	VHPZ00000000
E1336	X	X	X	X	X	X	X	X		X	Cambodia	2010	ND	III	LZSJ00000000
1242461	X	X	X	X	X	X	X	X		X	Mozambique	2012	human	III	LZLZ00000000
CT6	X	X	X	X	X	X	X	X		X	USA	2014	soil	III	NZ_CP011269.1
E190	X	X	X	X	X	X	X	X		X	Cambodia	2010	ND	III	LZIW00000000
NCTC1542	X	X	X	X	X	X	X	X		X	ND	1923	fish	III	UGQY00000000
E1141	X	X	X	X	X	X	X	X		X	Cambodia	2010	ND	III	LZIV00000000
isolate_1	X	X	X	X	X	X	X	X		X	China	2013	human	III	JAAZWN000000000
JCM6368	X	X	X	X	X	X	X	X		X	Japan	2015	ND	III	BCSZ00000000
isolate_2	X	X	X	X	X	X	X	X		X	China	2013	human	III	JAAZWM000000000
E3337	X	X	X	X	X	X	X	X	X	X	Cambodia	2011	ND	III	LZKO00000000
1089381.4	X	X	X	X	X	X	X	X		X	Mozambique	2012	human	III	LZLO00000000
7G	X	X	X	X	X	X	X	X		X	Brazil	2020	cat	III	JAEQRQ000000000
ATCC 6841	X	X	X	X	X	X	X	X		X	USA	1938	human	III	CP014258.1

The *in silico* inference of the resistome was performed using CARD, resulting in the identification, in all genomes of clusters I, II and III, of the followed genes: *aph* (aminoglycoside O-phosphotransferase), *aac* (aminoglycoside acetyltransferase), *arr* (NAD^+^ rifampin ADP-ribosyltransferase), *bla*F (beta-lactamase), *erm* (23S ribosomal RNA methyltransferase), *rbp*A (RNA polymerase-binding protein), *rox* (rifampin monooxygenase) and *tap* (multidrug efflux pump). Each of these genes was found in the same chromosomal location, with no evidence of association with mobile platforms, suggesting the constitutive nature of this resistome in this species. In addition to this *in silico* analysis and to gain some insights into the functionality of these antibiotic resistance genes (ARGs), we performed *in vitro* analyses with *M. fortuitum* 7G strain, defining the minimum inhibitory concentration (MIC) for the antibiotic classes represented in the resistome. The *M. fortuitum* 7G strain showed high resistance rates to macrolides (azithromycin > 256 µg/mL and clarithromycin > 32 µg/mL), aminoglycosides (streptomycin > 32 µg/mL and tobramycin > 32 µg/mL), carbapenem (meropenem > 32 µg/mL), cephalosporins (cefalotin > 256 µg/mL and cefepime > 256 µg/mL) and rifampicin > 32 µg/mL. In fact, Nash et al.^21^ observed that *M. fortuitum* strains were naturally resistant to macrolides and that this resistance would be associated with the *erm* gene. Erm belongs to a diverse family of proteins encoded by a heterogeneity of alleles, some of them (*erm*37-41*)* intrinsically associated with *Mycobacteriaceae* species. Here, *erm*39 was identified in all *M. fortuitum* genomes and its functionality was demonstrated in *M. fortuitum* 7G, as it had already been shown in the CT6 strain.^21^ The *aph*(*3*”)*-*Ic gene was identified for the first time in an environmental *M. fortuitum* strain, being involved in molecular mechanisms of streptomycin resistance in some *Mycobacteriaceae* and *Streptomyces*.^22^ It is worth noting the identification in all genomes of a set of determinants, related to different resistance mechanisms, associated with the rifamycin class of antibiotics. The *arr*, *rbp*A, *rox* and *tap* genes may impact resistance to rifampicin,^23^ ^,^ ^24^ a first-line drug that has been used to treat *Mycobacterium tuberculosis* infections for more than half a century. We then experimentally determined the activity of some of these genes (*arr* and *rox*) carried by the *M. fortuitum* 7G strain*.* Based on *arr* and *rox* genes cloning and transformation in heterologous system, it was demonstrated that this *arr* allele was associated with a high rate of resistance (32 µg/mL), while *rox* did not improve the *E. coli* rifampicin MIC, indicating that this Arr offers resistance to rifampin for *M. fortuitum* 7G strain. Besides the conservative *M. fortuitum* resistome, a class 1 integron carrying a qac/sul1 gene cassette was identified in a genome (E3337) from cluster III (Table) and, therefore, this would be the first evidence of this genetic element of resistance in *Mycolicibacterium*. Class 1 integron is a genomic platform in which antibiotic resistance genes are acquired and expressed, contributing to the emergence of resistance in a one-step fashion,^25^ therefore, it represents a possibility to increase the resistance spectrum in one generation. In general, in bacteria, plasmids are another genetic element strongly associated with the bacterial resistome, but particularly for *M. fortuitum*, its resistome was entirely associated with the chromosomal genomic context. In fact, a previous survey of *Mycolicibacterium* mobilome showed an 8 kb non-mobilisable plasmid, without association to any ARG, shared by two *M. fortuitum metagenomes* (SCH6189132/cluster II/South Africa and MTB7/cluster III/Morocco).^18^

The *M. fortuitum* virulome was accessed using ABRicate based on Virulence Factor Database. Four genes were identified in all genomes: *icl*, *ide*R, *pho*P, and *rel*A (except *rel*A in GA-0871). These genes were associated with stress response, persistence, and iron uptake, indirectly impacting the virulence. In addition, we also searched for the T7SS, which is the only specialised secretion system of these organisms. T7SS is encoded by six paralogous chromosomal loci (ESX-1, -2, -3, -4, -4 bis, and -5) and has been associated with several functions, including virulence.^26^ ^,^ ^27^ All *M. fortuitum* genomes had ESX-1, ESX-3, ESX-4 and ESX-4-bis Type VII secretion systems, some of which play an essential role in *Mycobacterium* virulence, nutrient uptake and conjugation.^26^ ^,^ ^27^

Here, the ubiquitous *M. fortuitum* bacterium proved to be a reservoir of an expressive intrinsic resistome and virulome, despite the spatiotemporal diversity of the strains, which indicates a constitutive trait of the species that may contribute to its success as a global opportunist pathogen.
